# Exceptional preservation reveals gastrointestinal anatomy and evolution in early actinopterygian fishes

**DOI:** 10.1038/srep18758

**Published:** 2016-01-06

**Authors:** Thodoris Argyriou, Marcus Clauss, Erin E. Maxwell, Heinz Furrer, Marcelo R. Sánchez-Villagra

**Affiliations:** 1 Paleontological Institute and Museum, University of Zurich, 8006 Zurich, Switzerland; 2 Clinic for Zoo Animals, Exotic Pets and Wildlife, Vetsuisse Faculty, University of Zurich, 8057 Zurich, Switzerland; 3 Stuttgart State Museum of Natural History, 70191 Stuttgart, Germany

## Abstract

Current knowledge about the evolutionary morphology of the vertebrate gastrointestinal tract (GIT) is hindered by the low preservation potential of soft tissues in fossils. Exceptionally preserved cololites of individual †*Saurichthys* from the Middle Triassic of Switzerland provide unique insights into the evolutionary morphology of the GIT. The GIT of †*Saurichthys* differed from that of other early actinopterygians, and was convergent to that of some living sharks and rays, in exhibiting up to 30 turns of the spiral valve. Dissections and literature review demonstrate the phylogenetic diversity of GIT features and signs of biological factors that influence its morphology. A phylogenetically informed analysis of a dataset containing 134 taxa suggests that body size and phylogeny are important factors affecting the spiral valve turn counts. The high number of turns in the spiral valve of †*Saurichthys* and some recent sharks and rays reflect both energetically demanding lifestyles and the evolutionary histories of the groups.

The anatomy of the vertebrate gastrointestinal tract (GIT) reflects many aspects of organismal biology, including diet and feeding habits and hence trophic position, nutrient uptake capabilities, osmoregulation and metabolism^1^,^2^. Although a broad evolutionary perspective on the digestive system has been achieved by studying the distribution of the GIT morphologies in extant vertebrates^1^,^2^,^3^, much information is missing due to the vast proportion of vertebrate animals that are now extinct. This is especially true for actinopterygians (ray-finned fishes), the most speciose group of vertebrates^4^,^5^. Whereas GIT diversity of derived actinopterygians (teleosts) is well documented, this is not the case for the non-teleostean actinopterygians, which are represented in the modern fauna by a few depauperate lineages: bichirs and reedfish (two genera and 16 species), sturgeons and paddlefishes (five genera and 27 species), gars (two genera and seven species) and the bowfin (one species)^4^. These taxa exhibit plesiomorphic GIT morphologies, including the presence of a spiral valve in the posterior part of the intestine (also known as the spiral or valvular intestine) that are reminiscent of those seen in living chondrichthyans and differ from those of teleosts^2^,^3^,^6^.

The spiral valve is formed by the intestinal mucosa and submucosa and resembles a spiral staircase extending along part of the length of the posterior mid-gut^3^,^6^. This structure differentiates in ontogeny as an invagination of the intestinal epithelium. The resulting crest, due to significant posteroanterior growth, twists around the median axis of the intestine forming successive spirals^3^,^6^,^7^. In several taxa, the initial crest wraps around the median axis of the intestine forming the “scroll valve”^3^,^6^,^7^. A spiral or scroll valve in the posterior part of the intestine is a plesiomorphic feature shared amongst chondrichthyans (including most “†acanthodians”), non-teleostean actinopterygians, non-tetrapod sarcopterygians (including extant lungfishes that possess a spiral valve and extant coelacanths that possess a scroll valve) and likely “†placoderms”^8^. The presence of a scroll valve has also been suggested for some early Paleozoic jawless vertebrates^9^,^10^ whereas modern lampreys, but not hagfishes, also exhibit a reduced spiral valve^3^,^6^. The spiral valve is clearly a character that appeared very early in the evolution of vertebrates.

Most paleontological perspectives on the vertebrate GIT are based on indirect evidence from fossilized faeces (coprolites)^11^ because the corresponding soft tissues are highly susceptible to decay and early loss during fossilization^12^. Stomach contents can provide some insights into GIT morphology but have been historically used for tracing feeding habits and trophic positions of extinct organisms^13^. In rare cases, internal casts of the GIT, deriving from fossilized chyme and/or faecal matter and termed “cololites”^14^, are preserved in the fossil record^11^. Cololite studies are scarce, mostly because GIT casts associated with taxonomically recognizable individuals are rarely preserved^8^,^15^ (see also Supplementary Table 1 for additional references). Cololites can reflect the gross morphology of the GIT and can provide insights into the biology and phylogeny of the studied organisms.

†Saurichthyids, known from latest Permian to Early Jurassic deposits worldwide, were highly specialized, predatory, non-neopterygian actinopterygians that shared an elongate body, an elongated preorbital region and posteriorly situated median fins^16^,^17^,^18^. Recent phylogenetic analyses consistently place †*Saurichthys*, the most salient genus of this “family”, as closely related to or part of the chondrostean clade and, often, close to the Triassic fish †*Birgeria*^19^,^20^,^21^,^22^ (but see ref. 19: Fig. 9A for an alternate placement of †*Saurichthys*).

As for most fossil organisms, little is known about the soft tissue anatomy of †*Saurichthys*^16^,^23^. Here, we provide the first detailed description of cololites from Middle Triassic species of †*Saurichthys* that constitute a rare and key source of data for studying the evolution of the GIT in early actinopterygians and fishes in general. The striking dissimilarity of the GIT morphology of †*Saurichthys*, and markedly that of its spiral intestine, to that of extant actinopterygians raises some paleobiological questions. We review the distribution of different morphologies of the GIT across extant and extinct fishes (including elasmobranchs, sarcopterygians and actinopterygians) in order to trace factors including body size, diet, lifestyle and phylogeny that may correlate with different GIT morphologies.

## Results

### †*Saurichthys costasquamosus*

**Figure 1:**
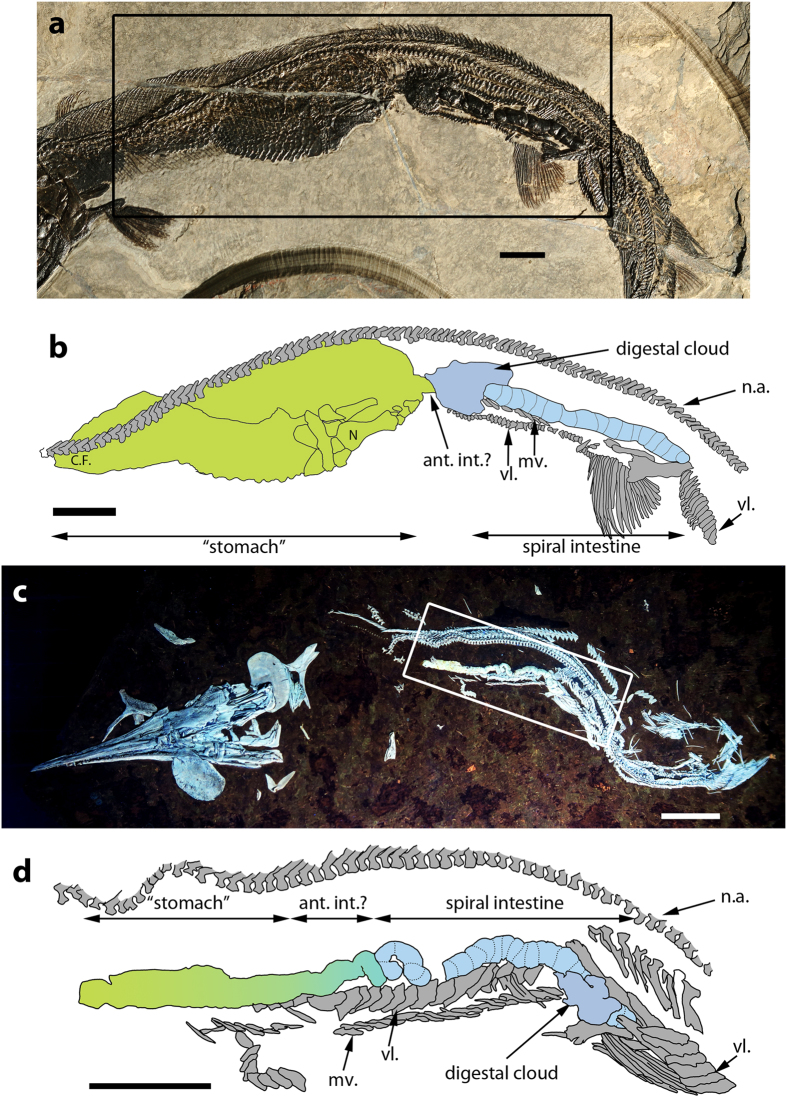
†*Saurichthys* specimens with preserved GIT casts. (**a**) †*Saurichthys costasquamosus* (MCSN 5696) with undigested actinopterygian prey (cf. †*Luganoia*) followed by a three dimensional spiral cololite. The area of interest is delineated by a box; (**b**) Interpretative drawing of the area of interest of the previous specimen. Scales of the midlateral row were omitted; (**c**) †*Saurichthys macrocephalus* (PIMUZ T 3916), photographed under UV light, with a two dimensional cololite present, extending from the stomach to the spiral intestine. The area of interest is delineated by a box; (**d**) interpretative drawing of the area of interest around the cololite. Abbreviations are as follows: ant.int.: anterior intestine; C.F.: caudal fin of the contained prey; mv.: medioventral scale row; N.: neurocranium of the contained prey; n.a.: neural arch-like elements; vl: ventrolateral scale row. All scale bars equal 1 cm.

Specimen MCSN 5696 (Fig. 1a,b) is an almost complete individual, only missing the anterior half of its rostrum. Total Length (TL) is slightly over 30 cm and thus smaller than the maximum known size for this species (up to 83 cm)^23^. Fossilized gut contents span almost the entire Abdominal Cavity Length (ACL). A complete individual of an early actinopterygian (cf. †*Luganoia*) is preserved as undigested prey in the abdominal cavity just posterior to the head. The prey occupies almost 40% of the ACL (25 vertebral segments^16^), was swallowed head first, and is arranged in an almost straight, uncoiled manner, reflecting the anatomy of the containing GIT chamber. The contained prey, due to its size, bulges out to the venter of the abdominal cavity of the predator. The distensibility of this GIT segment suggests that it is a true straight stomach rather than a pseudogaster (stomach-like thickening of the midgut seen in some agastric species)^2^,^6^,^13^.

Posterior to the head of the prey fish, the GIT chamber tapers and leads to an amorphous digestal cloud that corresponds topologically to the pyloric caeca. However, this structure does not exhibit any morphological (e.g., vermiform) patterns expected of pyloric caeca. We attribute its formation to tearing of the anterior intestine. Posterior to the digestal cloud, the substantial part of the three-dimensional cololite is observed. It measures 36.7 mm in length (23.3% of the ACL), 3.2 mm in height and spans 15 vertebral segments (30 neural arch-like elements^16^). The surface of the cololite was secondarily smoothened but several visible constrictions indicate the presence of a spiral valve that formed more than 17 turns. No gut infilling was preserved in the area between the end of the cololite and the anal opening. At least part of this empty area was presumably occupied by the rectum.

### †*Saurichthys macrocephalus*

The body of PIMUZ T 3916 is coiled in an S-shape and its head is detached^23^ (Fig. 1c). The TL is approximately 24 cm and, thus, smaller than the maximum TL for this species (66 cm, PIMUZ T 5631). Much of the GIT is well-preserved as a flattened white ribbon within the abdominal cavity^23^ (Fig. 1c, S1). Visible divisions of the GIT include the putative stomach, a short anterior intestine and part of the spiral intestine (Fig. 1d). The posterior end is obstructed by the pelvic bones, the ventrolateral scale row, and by a small cloud of faecal matter that likely escaped from the intestine after the latter was ruptured.

We refer to the straight and somewhat thickened part of the GIT, connecting the oesophagus to the anterior intestine, as the “stomach”. The absence of any sign of a pyloric valve, separating the “stomach” from the intestine, allows us to only tentatively identify a division between the two (Fig. 1c,d, S1). The preserved part of the “stomach” measures 15.3 mm, it spans ~16 neural arch-like elements (~eight vertebral segments^16^) and is straight, without an externally differentiated cardiac and pyloric part. The preserved segment seems to correspond to less than half of the organ’s length. The height of the organ is approximately 2.8 mm for most of its length but gradually tapers near the presumed transition to the anterior intestine.

The region we identify as the anterior intestine maintains a constant height, between 1.3 and 1.6 mm, along its length (Fig. 1c,d). The observed coil must have formed post mortem, due to elastic recoil of the GIT after the detachment of the head. The spiral intestine exhibits a larger diameter than the anterior intestine, with its depth reaching 1.9 mm. Approximately 17 constrictions on the preserved part of the spiral intestine correspond to spiral valve turns. The caudal part of the cololite is partially obscured by a digestal cloud and skeletal elements. The total turn count is estimated to have been comparable to that of †*S. paucitrichus* (see below).

### †*Saurichthys paucitrichus*

**Figure 2:**
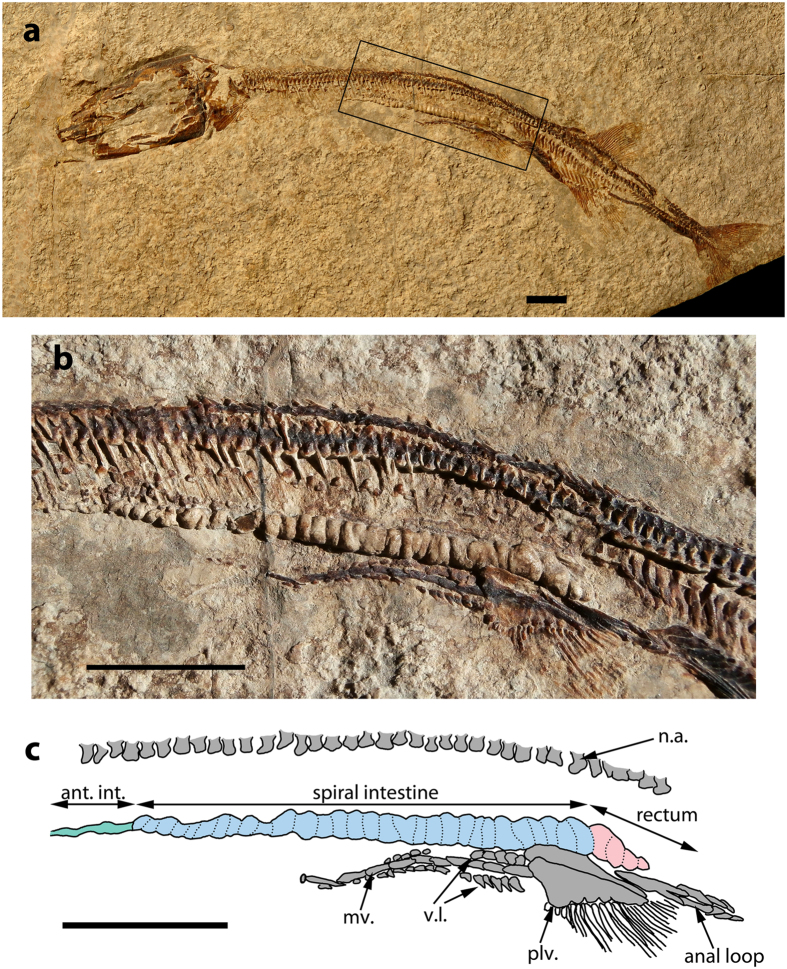
†*Saurichthys paucitrichus* with preserved GIT cast. (**a**) †*Saurichthys paucitrichus* (PIMUZ T 59) with a three dimensional intestinal cololite preserved *in situ*, the area of interest is delineated by a box; (**b**) Detail of the area of interest containing the spiral cololite in the previous specimen; (**c**) Interpretative drawing of the spiral cololite of the previous specimen. Abbreviations are as follows: ant.int.: anterior intestine; mv.: medioventral scale row; n.a.: neural arch-like elements; plv.: pelvic bone; vl: ventrolateral scale row. All scale bars equal 1 cm.

The specimen (PIMUZ T 59) has an estimated TL of 21.5 cm and ACL of 7.5 cm and exhibits a well-preserved, three-dimensional cololite of the post-gastric portion of the GIT (Fig. 2a–c). This corresponds to part of the anterior intestine, which is uncoiled, the complete spiral intestine and likely the cranial tip of the rectum. The cololite’s longitudinal axis is straight and runs parallel to the notochord, along the ventral part of the abdominal cavity. The anterior part of the cololite corresponds to part of the non-spiral anterior intestine and measures 3.9 mm in length (19% of the ACL), 0.6 mm in height and spans approximately three vertebral segments (six neural arch-like elements^16^).

The largest portion of the cololite is 29 mm in length (38.7% of the ACL), corresponds to the spiral intestine, forms 30 turns and spans approximately 27 to 28 neural arch-like elements (14 vertebral segments^16^). When viewed from anterior, the spiral part of the cololite exhibits a counter-clockwise spiral coiling pattern. The valvate portion gradually increases in height to 2 mm. The posterior-most part of the cololite tapers off before reaching the cloaca, which is delineated by the scales of the mid-ventral scale row that form a loop around the cloaca (anal loop). The individual turns are tightly packed and maintain a relatively constant width of 0.8–1 mm. The cranial portion of the spiral cololite indicates that the fecal ribbon wrapped around a median axis (typhlosole in life) without forming overlapping cones. This suggests that the radius of the mucosal folds was not larger than that of the intestinal casing and is similar to Parker’s “type B”^24^.

A thickening and deformation of the cololite is visible at the level of the 30th turn of the spiral valve. The last 4 mm of the cololite, including three to four additional turns, were likely preserved inside the rectum. The rectum measures 7.5 mm or 10% of the ACL.

### Comparisons with other taxa

**Figure 3:**
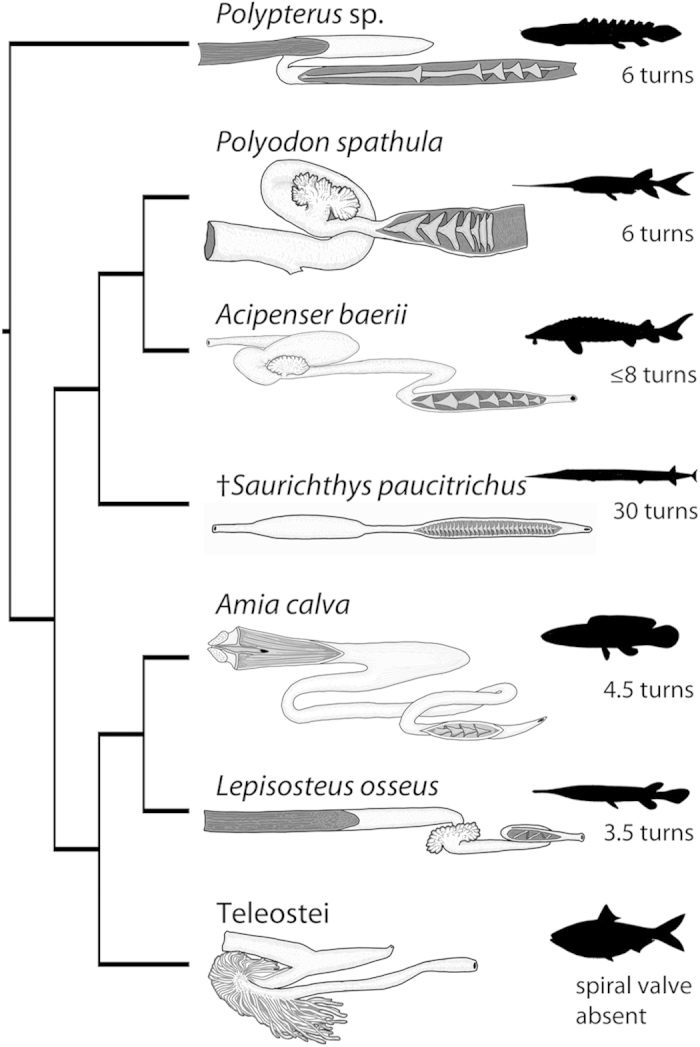
Phylogenetic framework of GIT morphology and spiral valve turn counts of actinopterygians, including †*Saurichthys paucitrichus.* Phylogenetic hypothesis based on refs 5,20. Interpretative drawings of GITs of *Polypterus*, *Polyodon spathula*, *Lepisosteus osseus* and the teleost *Alosa* redrawn and modified from ref 27. The interpretative drawing of the *Amia calva* GIT is redrawn and modified from ref 7. The drawings of the *Acipenser baerii* and †*S. paucitrichus* GITs are based on our observations. All drawings depict the GIT in ventral view with foregut to the left and hindgut to the right.

One striking difference between GIT anatomy in †*Saurichthys* and extant non-teleostean actinopterygians is the linear arrangement of the GIT in the former. All known non-teleostean actinopterygians show either an S-shaped arrangement (*Polypterus*, *Acipenser*, *Polyodon*) or a more complex arrangement consisting of two intestinal loops (*Lepisosteus*, *Amia*) (Fig. 3, S4,5).

The stomach of basal actinopterygians shows an array of forms. In extant lepisosteiforms^25^ and in †*Saurichthys* the stomach is straight, tube-shaped, and the cardiac and pyloric regions cannot be macroscopically differentiated. However, in extant lepisosteiforms the stomach is easily distinguishable from the intestine in having a larger diameter and clearly tapering caudal end^25^. In †*Saurichthys macrocephalus* (Fig. 1c) there is no clear constriction between the stomach and the intestine that could correspond to a pyloric valve. Therefore, the lack of a stomach cannot be ruled out. This agastric condition occurs in some extant teleosts but is unknown in extant non-teleostean actinopterygians^2^,^6^,^13^. Stomachs of other extant non-teleostean actinopterygians exhibit macroscopically recognizable cardiac and pyloric portions. In *Polypterus* the stomach is Y-shaped (cecal [sic] type^26^) forming posterior caecum-like structures that increase the storage capabilities of the organ^27^ (Fig. 3.). In acipenseriforms (S4,5) and *Amia* the stomach is U-shaped^25^,^28^,^29^,^30^ (Fig. 3).

In †*Saurichthys,* the anterior intestine appears to be short and straight (Figs 1c,d and 2b,c). Short anterior intestines are also seen in *Polypterus* and *Polyodon* (S5), but they form a curvature before connecting to the spiral intestine^27^,^30^. In acipenserids the anterior intestine is slightly longer and is arranged in an S-shaped manner^29^,^31^(S4). The gars and the bowfin have longer anterior intestines that are more coiled than in more basal taxa, forming two loops^25^,^28^.

†*Saurichthys* deviates from the common conicospiral condition (mucosa forming a median typhlosole and overlapping cones) seen in living non-teleostean actinopterygians, approaching the ring-type condition seen in some extant elasmobranchs^2^,^6^ and some fossil †pachycormids^15^,^26^,^32^. The most striking aspect is the number of turns of the spiral valve (up to 30). Extant non-teleostean actinopterygians exhibit between 3.5 to 8 turns^25^,^27^,^28^,^29^,^30^,^31^,^33^ (Fig. 3; Supplementary Table 1). Several Mesozoic actinopterygians exhibited a low spiral valve turn count, comparable to that of extant species^15^, but the †pachychormid †*Asthenocormus titanius* is a notable exception, with a turn count >70^15^. Some 17 turns were also described for intestines of †*Amblysemius pachyurus*^15^. The rectum was short in †*Saurichthys*, comparable to extant non-teleostean actinopterygians.

**Figure 4:**
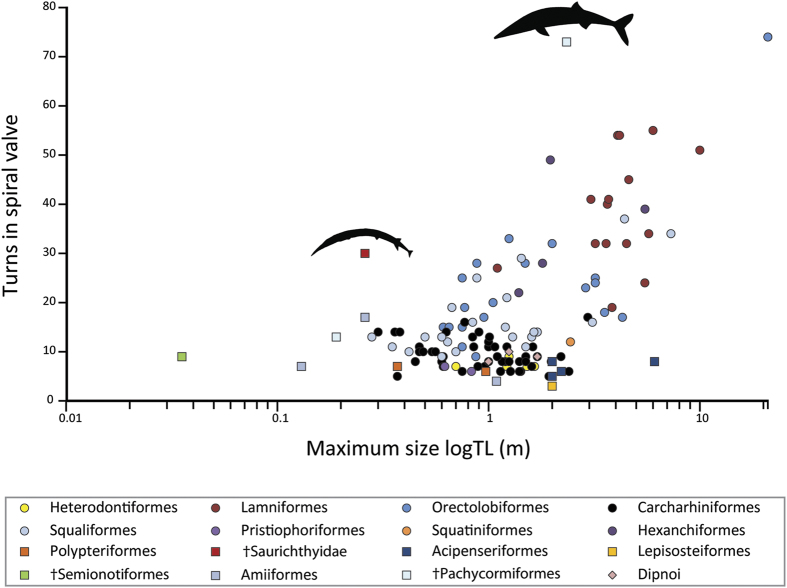
Relationship between maximum body size (logTL) and maximum spiral valve turn counts. Data and references in ST1. Different fish groups (“orders”) are color coded. Elasmobranchs: The general trend of turn increase with body size is evident. However, the constancy or decreased variability of turn counts within groups is also marked. †*Saurichthys paucitrichus* (PIMUZ T 59, thinner black outline) and †*Asthenocormus titanius* (thicker black outline) plot as outliers, exhibiting a much higher turn count than all extant osteichthyans and elasmobranchs of similar size. It should be noted that extant osteichthyans tend to exhibit fewer turns than most elasmobranchs despite achieving moderate body sizes.

A statistical evaluation of the association of maximum body length and maximum turns of the spiral valve (Supplementary Table 1) indicates a significant increase in the number of turns with increasing body size (Fig. 4), both in Ordinary Least Squares (OLS, without accounting for the phylogenetic structure of the dataset) and in Phylogenetic Generalized Least Squares (PGLS, i.e. with accounting for the phylogenetic structure of the dataset), in the complete dataset and the dataset of sharks only (Table 1). In both datasets, the phylogenetic signal λ is not different from 1, and the PGLS model has a lower AIC than the OLS model, indicating that there is phylogenetic structure in the dataset. Visually, this structure corresponds to the statistical result that, when accounting for phylogeny, the increase in the number of turns with body size is much less steep, and because the number of turns is taxon-specific, the confidence interval of the intercept increases (Table 1).

## Discussion

†*Saurichthys* possessed a short GIT that spanned the length of the abdominal cavity and consisted of a straight stomach or, less likely, a pseudogaster, a short anterior intestine and a markedly developed spiral intestine, all arranged in a linear manner. The presence of a straight stomach is a rare condition found in some carnivorous actinopterygian fish clades (convergently evolved in lepisosteiforms and esociforms) and is considered a precursor of stomach loss^2^. Stomach loss occurred independently in lampreys, chimaeras and several teleost groups including cypriniforms, beloniforms, labrids, and tetraodontiforms, among others^2^,^6^.

†*Saurichthys* swallowed their prey whole and unchewed^23^ (Fig. 1a,b; S1). In addition, the intestinal contents in †*Saurichthys* are homogeneous and do not exhibit macroscopically recognizable bony elements. These facts suggest a reliance on chemical digestion that probably involved a true stomach, rendering an agastric condition in †*Saurichthys* unlikely. The apparent absence of any trace of pyloric caeca can be attributed to either incomplete preservation or to an actual absence of pyloric caeca in †*Saurichthys*. The second possibility is more likely because extant agastric actinopterygians as well as actinopterygians with straight stomachs do not possess pyloric caeca^34^. Gars deviate from this pattern and exhibit well-developed caeca^25^ (TA pers. obs. on *Lepisosteus osseus*), as do most carnivorous actinopterygians^34^.

A spirally coiled portion indicating the presence of a spiral valve with a very high turn count is a remarkable feature of †*Saurichthys* cololites. The main function of the spiral valve is to increase the length of the intestinal lumen, and therefore maximize the effective surface for absorption and enzymatic digestion while maintaining a relatively short intestinal casing^2^,^6^. The spiral valve can increase the intestinal surface threefold (ring-type valves without a median typhlosole) to sixfold (strongly conicospiral valves)^7^. Fishes with a spiral valve have shorter overall intestinal lengths than other species^35^, conserving space in the abdominal cavity for other purposes (such as developing embryos)^36^. These two features characterize †*Saurichthys*. In contrast, teleosts, which do not possess a spiral intestine, increase intestinal surface area by increasing total length of the intestine, which subsequently forms loops and becomes tightly packed within the abdominal cavity^2^,^6^, or through the development and multiplication of pyloric caeca^34^.

In teleosts, an increase in intestinal length is associated with a transition from carnivorous to omnivorous or more herbivorous diets^35^,^37^,^38^. Indeed, diet has been historically considered as the prevailing factor influencing the number of spiral valve turns^36^,^39^,^40^,^41^. For instance, the “voracious” and often pelagic predators (such as most lamniforms and hexanchiformes) and the planktivorous chondrichthyans (such as *Rhinchodon typus*, *Cetorhinus maximus, Megachasma pelagios* and the mobulid rays) exhibit ring-type valves with very high turn counts^39^ (Supplementary Table 1). In contrast, the only extant non-teleostean actinopterygian with a planktivorous diet, the Mississippi paddlefish (*Polyodon spathula*), does not exhibit a similar increase in spiral valve turns, but the last three turns become closely stacked to resemble the ring-type condition (Fig. 3). However, the functional significance of this close stacking of the spiral valve turns is still unclear.

Being large is common to pelagic top predators and planktivores. Given the overarching relevance of body size for dimensions in anatomical structures and biology^42^,^43^, we examined its relation to spiral valve turns for the first time. Our analysis suggests that even though there is distinct phylogenetic inertia with respect to number of turns across a range of body sizes, larger animals have a higher number of turns when corrected for relatedness. Limited evidence suggests that the number of turns in the spiral valve is ontogenetically stable, suggesting little influence of growth on this characteristic^44^ (TA pers. obs. on juveniles of *Acipenser gueldenstaedtii*).

Among fishes with similar diets, metabolism and activity levels have also been suggested to correlate with intestinal length. For example, active pelagic carnivores (e.g., tunas) tend to have longer intestines than ambush predators (e.g., pikes)^40^,^45^. An analogous condition might apply to recent elasmobranchs. For example, the pelagic and active lamniforms exhibit high spiral valve turn counts in comparison with more benthic taxa like some orectolobiforms or some carcharhiniforms^39^ (Supplementary Table 1). However, a reliable classification for activity level or metabolic rate is not yet available and will be required to test this hypothesis. Phylogeny also plays an important role in understanding the variation in spiral valve counts in fishes, with closely related species and genera tending to exhibit similar turn counts^39^,^46^. This applies generally to extant taxa despite fluctuations in size and different trophic niches, rendering functional interpretations questionable if not controlled for relatedness.

The high number of spiral valve turns in both †*Saurichthys paucitrichus* and †*Asthenocormus titanius* places these species as outliers to the common pattern of extant fishes (Fig. 4). The biology of †*Saurichthys* provides clues for the potential role of a well-developed spiral valve. First, †*Saurichthys* might have been particularly active, or had an unusually high metabolism^40^. However, †*Saurichthys* has been described as an ambush predator, likely incapable of rapid sustained swimming^16^,^23^,^47^,^48^, which is incongruent with an energetically demanding lifestyle^49^. Viviparity^23^,^50^ and potential maternal provisioning could only partially explain such an increase in energetic demand. Alternatively, given the relationship between spiral valve turns and body size, this position indicates, if maturity is assumed (Supplementary Note 1), a secondary dwarfed form that retained a characteristic typical of a larger ancestor. For instance, the closely related and sympatric †*S. costasquamosus* attained a total length of ~85 cm (PIMUZ T 1275)^23^, and the Early Triassic †*S. dayi* exceeded 1.5 m in length^51^. Furthermore, the potential close phylogenetic proximity of †*Saurichthys* to the larger and more pelagic †*Birgeria*^19^,^20^,^21^,^22^ might also explain the high number of spiral valve turns in †*S. paucitrichus*. We therefore hypothesize that the presence of such well-developed spiral intestines is a plesiomorphic condition for †Saurichthyidae, retained in smaller species. The multi-valvate condition seen in †*Asthenocormus* invites a similar interpretation based on its sister taxon relationship with the emblematic giant †*Leedsichthys*^32^. The turn counts in the spiral valves of †saurichthyids and †pachycormids reveal that increased turn multiplication occurred, independently, at least twice in the evolutionary history of actinopterygians; once in the chondrostean clade and once on the teleost stem. Also, the independent occurrences of high turn counts in large chondrichthyans and actinopterygians constitute examples of broad evolutionary convergence and underline the potential functional relevance of this trait and its relationship to body size.

In conclusion, we emphasize the importance of investigating gastrointestinal contents in fossils, because they often reflect the morphology of the surrounding soft tissue and therefore provide information on palaeobiology and phylogeny that would otherwise remain elusive.

## Methods

### Locality information and specimens

The fossils treated here come from the Middle Triassic UNESCO World Heritage Site of Monte San Giorgio, Switzerland (Besano and Meride formations^52^). The Lagerstätte deposits exposed at the site are known for the exceptional preservation of delicate structures including embryos^23^,^50^ and soft tissues^16^,^23^. Several †*Saurichthys* specimens exhibit traces of fossilized digesta within their body cavities, but very few provide a clear and more complete picture of the GIT. This work focuses on three specimens: one †*Saurichthys paucitrichus* (PIMUZ T 59) from the Besano Formation (earliest Ladinian), one †*S. macrocephalus* (PIMUZ T 3916) and one †*S. costasquamosus* (MCSN 5696) from the overlying (early Ladinian) Meride Formation^23^,^47^. Additional information was extracted from other, less well-preserved specimens including: †*S. macrocephalus* (PIMUZ T 4106, S2); †*S. breviabdominalis* (PIMUZ T 890, S3); †*S. curionii* (PIMUZ T 5679, PIMUZ T 5684, PIMUZ T 5827) and †*Saurichthys* sp. (PIMUZ T 1768a,b).

We compared the morphology of the GIT of †*Saurichthys* to that of living bracketing or closely related actinopterygian taxa (Fig. 3). We dissected wet specimens including: *Polypterus* sp. (Z-M-UZH 140016, alcohol preserved, Zoological Museum, University of Zurich); *Acipenser baerii* and *A. gueldenstaedtii* (fresh juvenile and adult specimens donated by Frutigen AG and discarded after the dissection); *Polyodon spathula* (VIMS 12227, alcohol preserved, Virginia Institute of Marine Science); *Lepisosteus osseus* (VIMS 17602, alcohol preserved). Our observations were supplemented with data from the literature.

### Photography

Specimens were photographed under “normal” light. We experimented with an Ultraviolet (UV) hand lamp (230V, 50Hz, 40VA) in order to enhance the contrast and the visibility of the studied structures^53^ but, this produced adequate results only in the case of †*S. macrocephalus* (PIMUZ T 3916) from the Meride Fm., which is heavily phosphatized.

### Literature data

We collated literature data on recent and fossil fishes in order to explore the relationship of the number of turns in the spiral valve to body size (134 taxa, Supplementary Table 1). When the TL of fossil taxa was not readily available, we measured it from published figures. We tested the relationship between log-transformed maximum body length and log-transformed maximum number of turns of the spiral valve according to *log (number of turns)* *=* *a* *+* *b log (maximum Total Length)*, using Ordinary Least Squares (OLS) and Phylogenetic Generalized Least Squares (PGLS) in the whole dataset and in a taxonomic subset. For the PGLS analysis, we used a tree constructed based on a recent phylogeny of elasmobranchs^54^, to which several extant and extinct osteichthyan taxa, including †*Saurichthys paucitrichus*, were added (see Supplementary Note 2 for additional methods and references) while chimaeriforms and batoids were excluded (data on spiral valve morphology not readily available in the literature and/or body size not effectively explained by TL). Branch lengths of this modified tree were set to 1, because the resulting tree was not based on our own calculations of branch lengths after consistent use of the same characters. In contrast, the analysis for sharks alone included the original information on branch lengths^54^.

PGLS was used with Pagel’s λ^55^, estimated by maximum likelihood. λ can vary between 0 (no phylogenetic signal) and 1 (the observed pattern is predicted by the phylogeny; similarity among species scales in proportion to branch length)^55^. OLS and PGLS models were compared for goodness-of-fit using Akaike’s Information Criterion (AIC), with better-supported models having a lower AIC^56^. Statistical tests were performed in R 2.15.0^57^ using the packages caper^58^, and nlme^59^. We display results of both OLS and PGLS analyses, because a comparison of the respective results facilitates interpretation^60^, e.g. such as realizing whether accounting for phylogeny leads to a steeper or shallower relationship than expected from the raw data.

## Additional Information

**How to cite this article**: Argyriou, T. *et al.* Exceptional preservation reveals gastrointestinal anatomy and evolution in early actinopterygian fishes. *Sci. Rep.* **6**, 18758; doi: 10.1038/srep18758 (2016).

## Supplementary Material

Supplementary Information

## Figures and Tables

**Table 1:** Statistical analysis of the relationship between TL and spiral valve turn count of fishes.

Dataset	Statistics	λ (95% CI)	a (95% CI)	t	p	b (95% CI)	t	p	AIC
Including osteichthyans	OLS	(0)	12.4 (10.3;14.4)	47.161	<0.001	0.35 (0.24;0.47)	6.108	<0.001	34.408
(n = 134)	PGLS^1^	1.000 (0.963;NA)	10.9 (8.0;13.9)	5.809	<0.001	0.11 (0.01;0.21)	2.094	0.038	−44.016
Elasmobranchs only	OLS	(0)	12.5 (10.4;14.6)	50.558	<0.001	0.47 (0.35;0.58)	7.939	<0.001	−7.430
(n = 117)	PGLS^2^	0.982 (0.933;NA)	13.8 (11.5;16.2)	14.327	<0.001	0.19 (0.11;0.27)	4.630	<0.001	−153.952

Statistical analyses of the relationship between the maximum length of species (x) and the maximum number of turns in their spiral valve (y) according to y = a x^b^ (for analysis by linear regression, values were log-transformed), using Ordinary Least Squares (OLS) or Phylogenetic Generalized Least Squares (PGLS). Data from Supplementary Table 1.

NA not applicable.

^1^with branch lengths set to 1.0.

^2^including branch lengths.

## References

[b1] Stevens C. E. & Hume I. D. Comparative physiology of the vertebrate digestive system. (Cambridge University Press, 2004).

[b2] Wilson J. & Castro L. In The multifunctional gut of fish Vol. 30 (eds Grosell M., Farrell A. & Brauner C. ) Ch. 1, 1–55 (Academic Press, 2011).

[b3] Jacobshagen E. In Handbuch der vergleichenden Anatomie der Wirbeltiere Vol. 3 (eds Bolk L., Göppert E., Kallius E. & Lubosch W. ) Ch. IV, 563–724 (Urban and Schwartzenberg, 1937).

[b4] Nelson J. S. Fishes of the world. Fourth Edition, (John Wiley & Sons, 2006).

[b5] Near T. J. *et al.* Resolution of ray-finned fish phylogeny and timing of diversification. Proc. Natl. Acad. Sci. USA 109, 13698–13703 (2012). 22869754 10.1073/pnas.1206625109 PMC3427055

[b6] Harder W. Anatomy of fishes. 1–612 (E. Schweizerbart’sche Verlagsbuchhandlung, 1975).

[b7] Bertin L. In Traité de zoologie. Anatomie, systématique, biologie Vol. 13 (ed Grassé P. P. ) 1248–1302 (Masson et Cie éditeurs, 1958).

[b8] McAllister J. A. Phylogenetic distribution and morphological reassesment of the intestines of fossil and modern fishes. Zool. Jb. Anat. 115, 281–294 (1987).

[b9] Gilmore B. Scroll coprolites from the Silurian of Ireland and the feeding of early vertebrates. Palaeontology 35, 319–333 (1992).

[b10] Aldridge R. J., Gabbott S. E., Siveter L. J. & Theron J. N. Bromalites from the Soom Shale Lagerstätte (Upper Ordovician) of South Africa: Palaeoecological and palaeobiological implications. Palaeontology 49, 857–871 (2006).

[b11] Hunt A. P., Lucas S. G., Milàn J. & Spielmann J. A. In Vertebrate coprolites Vol. 57 (eds Hunt A. P., Milàn J., Lucas S. G. & Spielmann J. A. ) 1–24 (New Mexico Museum of Natural History & Science, 2012).

[b12] Sansom R. S., Gabbott S. E. & Purnell M. A. Atlas of vertebrate decay: A visual and taphonomic guide to fossil interpretation. Palaeontology 56, 457–474 (2013).

[b13] Viohl G. In Evolutionary paleobiology of behavior and coevolution (ed Boucot A. J. ) 287–303 (Elsevier, 1990).

[b14] Agassiz L. Recherches sur les poissons fossiles. Vol. 3 (Imprimerie de Petitpierre, 1833–1843).

[b15] Neumayer L. Vergleichend anatomische untersuchungen über den darmkanal fossiler fische. Abh. Bayer. Akad. Wiss. 29, 1–28 (1919).

[b16] Maxwell E. E., Furrer H. & Sanchez-Villagra M. R. Exceptional fossil preservation demonstrates a new mode of axial skeleton elongation in early ray-finned fishes. Nat. Commun. 4, 2570 (2013). 24096879 10.1038/ncomms3570

[b17] Maxwell E. E., Romano C., Wu F. & Furrer H. Two new species of *Saurichthys* (Actinopterygii: Saurichthyidae) from the Middle Triassic of Monte San Giorgio, Switzerland, with implications for character evolution in the genus. Zool. J. Linn. Soc. 173, 887–912 (2015).

[b18] Romano C., Kogan I., Jenks J., Jerjen I. & Brinkmann W. *Saurichthys* and other fossil fishes from the late Smithian (Early Triassic) of Bear Lake County (Idaho, USA), with a discussion of saurichthyid palaeogeography and evolution. Bull. Geosci. 87, 543–570 (2012).

[b19] Coates M. I. Endocranial preservation of a Carboniferous actinopterygian from Lancashire, UK, and the interrelationships of primitive actinopterygians. Philos. Trans. R. Soc. London Biol. 354, 435–462 (1999).

[b20] Gardiner B., Schaeffer B. & Masserie J. A review of the lower actinopterygian phylogeny. Zool. J. Linn. Soc. 144, 511–525 (2005).

[b21] Wu F., Chang M.-m., Sun Y. & Xu G. A new saurichthyiform (Actinopterygii) with a crushing feeding mechanism from the Middle Triassic of Guizhou (China). PloS one 8, e81010 (2013). 24324657 10.1371/journal.pone.0081010 PMC3852010

[b22] Xu G.-H., Gao K.-Q. & Finarelli J. A. A revision of the Middle Triassic scanilepiform fish *Fukangichthys longidorsalis* from Xinjiang, China, with comments on the phylogeny of the Actinopteri. J. Vertebr. Paleontol. 34, 747–759 (2014).

[b23] Rieppel O. Die Triasfauna der Tessiner Kalkalpen xxv: Die Gattung *Saurichthys* (Pisces, Actinopterygii) aus der mittleren Trias des Monte San Giorgio, Kanton Tessin. Schweiz. Palaeontol. Abh. 108, 1–103 (1985).

[b24] Parker T. J. V. On the intestinal spiral valve in the genus Raia. Zool. Soc. Lond. Trans. 11, 49–61 (1880).

[b25] Macallum A. B. Alimentary canal and pancreas of *Acipenser*, *Amia*, and *Lepidosteus*. J. Anat. Physiol 20, 604–636 (1886). PMC1288594 17231649

[b26] Arratia G. & Schultze H.-P. In Mesozoic fishes Vol. 5 (eds Arratia G., Schultze H.-P. & Wilson M. ) 87–120 (Dr. Friedrich Pfeil, 2013).

[b27] Gegenbaur C. Vergleichende Anatomie der Wirbelthiere mit Berücksichtigung der Wirbellosen. Vol. 2 696 (Verlag von Wilhelm Engelmann, 1901).

[b28] Hilton W. A. On the intestine of *Amia calva*. Am. Nat. 34, 717–735 (1900).

[b29] Buddington R. K. & Christofferson J. P. Digestive and feeding characteristics of the chondrosteans. Environ. Biol. Fish. 14, 31–41 (1985).

[b30] Weisel G. F. Anatomy and histology of the digestive system of the paddlefish (*Polyodon spathula*). J. Morphol. 140, 243–255 (1973). 10.1002/jmor.1051400209 30332857

[b31] Weisel G. F. Histology of the feeding and digestive organs of the shovelnose sturgeon, Scaphirhynchus platorynchus. Copeia 1979, 518–525 (1979).

[b32] Liston J. In Mesozoic fishes 4. Homology and phylogeny (eds Arratia G., Schultze H.-P. & Wilson M. V. H. ) 181–197 (Verlag Dr. Friedrich Pfeil, 2008).

[b33] Purser G. L. IV.—*Calamoichthys calabaricus* J. A. Smith. Part i. The alimentary and respiratory systems—concluded. Earth Environ. Sci. Trans. R. Soc. Edinb. 56, 89–101 (1929).

[b34] Buddington R. K. & Diamond J. M. Pyloric ceca of fish: A “new” absorptive organ. Am. J. Physiol. Gastrointest. Liver Physiol. 252, G65–G76 (1987). 10.1152/ajpgi.1987.252.1.G65 3812690

[b35] Karachle P. K. & Stergiou K. I. Gut length for several marine fish: Relationships with body length and trophic implications. Mar. Biodivers. Rec. 3, e106 (2010).

[b36] Wetherbee B. M., Cortés E. & Bizzarro J. J. In The biology of sharks and their relatives (eds Carrier J. C., Musick J. A. & Heithaus M. R. ) 239–264 (CRC Press, 2012).

[b37] Wagner C. E., McIntyre P. B., Buels K. S., Gilbert D. M. & Michel E. Diet predicts intestine length in lake Tanganyika’s cichlid fishes. Funct. Ecol. 23, 1122–1131 (2009).

[b38] Kramer D. & Bryant M. Intestine length in the fishes of a tropical stream: 2. Relationships to diet—the long and short of a convoluted issue. Environ. Biol. Fish. 42, 129–141 (1995).

[b39] Qingwen M. & Yuanding Z. A study of the spiral valves of chinese cartilaginous fishes. Acta Zool. Sin. 31, 277–284 (1985).

[b40] Buddington R. K., Krogdahl A. & Bakke-McKellep A. M. The intestines of carnivorous fish: Structure and functions and the relations with diet. Acta Physiol. Scand. 161, *Suppl.* 368, 67–80 (1997). 9421581

[b41] Holmgren S. & Nilsson S. In Sharks, skates, and rays: The biology of elasmobranch fishes (ed Hamlett W. C. ) 144–173 (The John Hopkins University press, 1999).

[b42] Calder W. Size, function and life history (Havard University Press, 1996).

[b43] Peters R. H. The ecological implications of body size. Vol. 2 (Cambridge University Press, 1986).

[b44] Neumayer L. Die Entwicklung des Darms von *Acipenser*. Acta Zool. 11, 39–150 (1930).

[b45] Suyehiro Y. A study on the digestive system and feeding habits of fish. Jpn. J. Zool. IX, 1–303 (1942).

[b46] White E. G. Interrelatonships of elasmobranchs with a key to the Order Galea. Bull. Am. Mus. Nat. Hist. 74, 25–138+151 tables (1937).

[b47] Rieppel O. A new species of the genus *Saurichthys* (Pisces: Actinopterygii) from the Middle Triassic of Monte San Giorgio (Switzerland), with comments on the phylogenetic interrelationships of the genus. Palaeontogr. Abt. A 221, 63–94 (1992).

[b48] Wu F., Sun Y., Xu G., Hao W. & Jiang D. New saurichthyid actinopterygian fishes from the Anisian (Middle Triassic) of southwestern China. Acta Palaeontol. Pol. 56, 581–614 (2011).

[b49] Fu S.-J. *et al.* The behavioural, digestive and metabolic characteristics of fishes with different foraging strategies. J. Exp. Biol. 212, 2296–2302 (2009). 19561220 10.1242/jeb.027102

[b50] Renesto S. & Stockar R. Exceptional preservation of embryos in the actinopterygian *Saurichthys* from the Middle Triassic of Monte San Giorgio, Switzerland. Swiss J. Geosci. 102, 323–330 (2009).

[b51] Mutter R. J., Cartanyà J. & Basaraba S. A. In Mesozoic fishes Vol. 4 (eds Arratia G., Schultze H.-P. & Wilson M. ) 103–127 (Verlag Dr. Friedrich Pfeil, 2008).

[b52] Furrer H. Der Monte San Giorgio im Südtessin-vom Berg der Saurier zur fossil-Lagerstätte internationaler Bedeutung. Njbl. natf. Ges. Zürich 206, 1–64 (Koprint, 2003).

[b53] Tischlinger H. & Arratia G. In Mesozoic fishes Vol. 5 (eds Arratia G., Schultze H.-P. & Wilson M. V. H. ) 549–560 (Verlag Dr. Friedrich Pfeil, 2013).

[b54] Naylor G. J. *et al.* In The biology of sharks and their relatives (eds Carrier J. C., Musick J. A. & Heithaus M. R. ) 31–56 (CRC Press, 2012).

[b55] Pagel M. Inferring the historical patterns of biological evolution. Nature 401, 877–884 (1999). 10553904 10.1038/44766

[b56] Burnham K. P. & Anderson D. R. Model selection and multimodel inference: A practical information-theoretic approach. (Springer Science & Business Media, 2002).

[b57] R Core Team (2013). R: A language and environment for statistical computing. R Foundation for Statistical Computing, Vienna, Austria. URL http://www.R-project.org/.

[b58] Orme D., Freckleton R., Thomas G., Petzoldt T., Fritz S., Isaac N. & Pearse W. (2012). Caper: comparative analyses of phylogenetics and evolution in R. Version 0.5. URL http://caper.r-forge.r-project.org/.

[b59] Pinheiro J., Bates D., DebRoy S., Sarkar D. & Core Team R (2011). Nlme: linear and nonlinear mixed effects models. R package version 3. URL http://cran.r-project.org/package=nlme.

[b60] Clauss M., Dittmann M. T., Müller D. W. H., Zerbe P. & Codron D. Low scaling of a life history variable: Analysing eutherian gestation periods with and without phylogeny-informed statistics. Mamm. Biol. 79, 9–16 (2014).

