# Supplementary material for: Exceptional preservation reveals gastrointestinal anatomy and evolution in early actinopterygian fishes
Source: Sci Rep. 2016 Jan 6;6:18758. doi: 10.1038/srep18758 (PMC4702121; doi:10.1038/srep18758)
Supplement: Supplementary Information [file srep18758-s1.pdf]

Supplementary information

Exceptional preservation reveals gastrointestinal anatomy and evolution in early actinopterygian fishes

Thodoris Argyriou, Marcus Clauss, Erin E. Maxwell, Heinz Furrer, and Marcelo R. Sánchez-Villagra

Supplementary Figure S1 | †*Saurichthys macrocephalus* (PIMUZ T 3916) detail of the preserved cololite, photographed under UV light. Scale bar equals 1 cm.

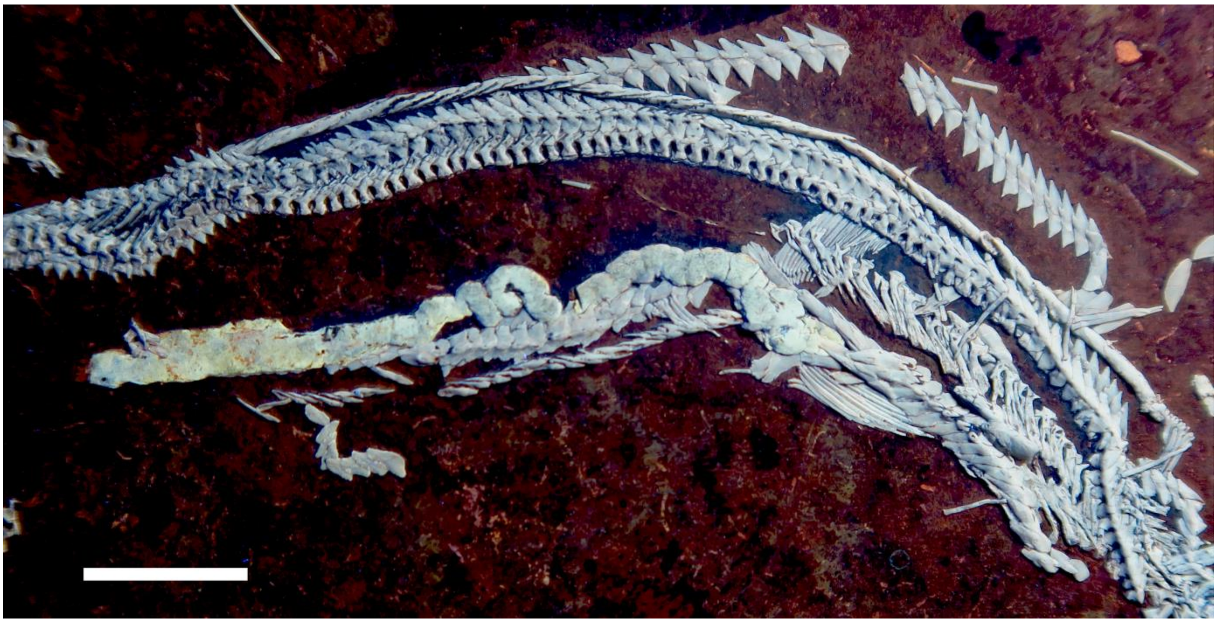

Supplementary Figure S2│ †*S. macrocephalus* (PIMUZ T 4106) with preserved straight stomach outline (stomach length is indicated by a double, black arrow) and a smaller *Saurichthys* specimen as undigested prey (N indicates the neurocranium of the prey). Scale bar equals 1 cm.

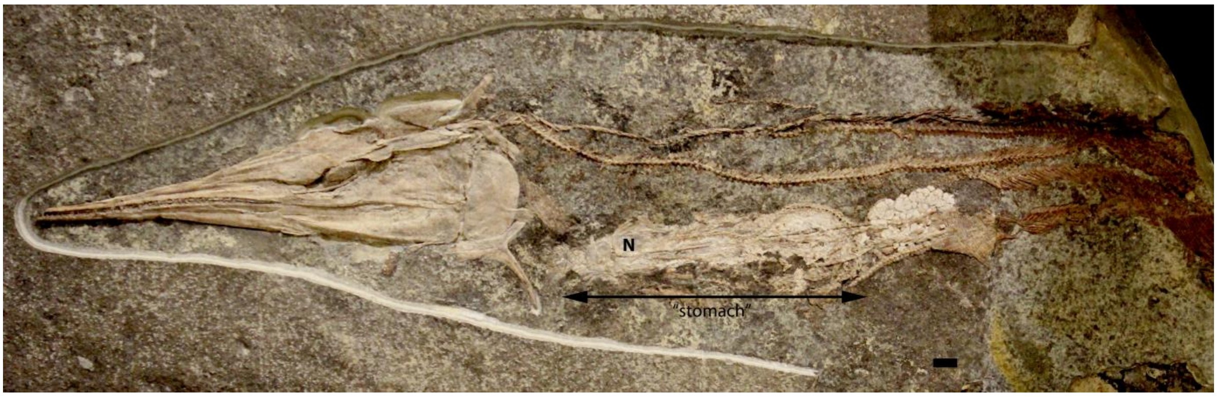

Supplementary Figure S3│ †*S. breviabdominalis* (PIMUZ T 890) exhibiting an incomplete spiral cololite. Scale bar equals 1 cm.

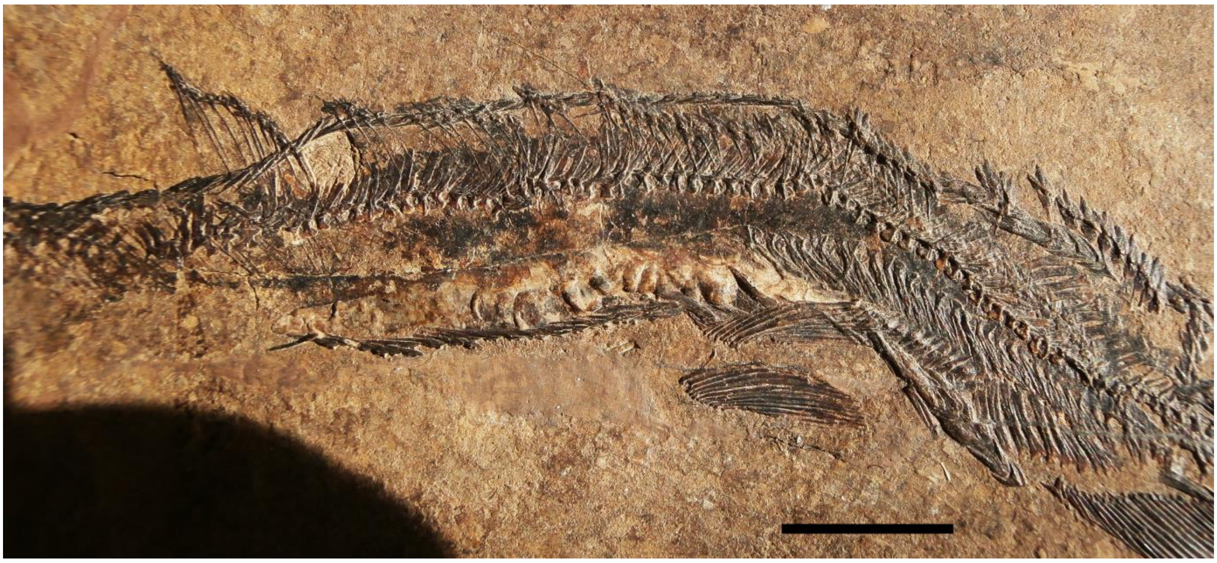

Supplementary Figure S4│ *Acipenser baerii* dissected GIT in ventrolateral view (top) and dissected spiral intestine (bottom), for comparison. Scale bars equal 1 cm.

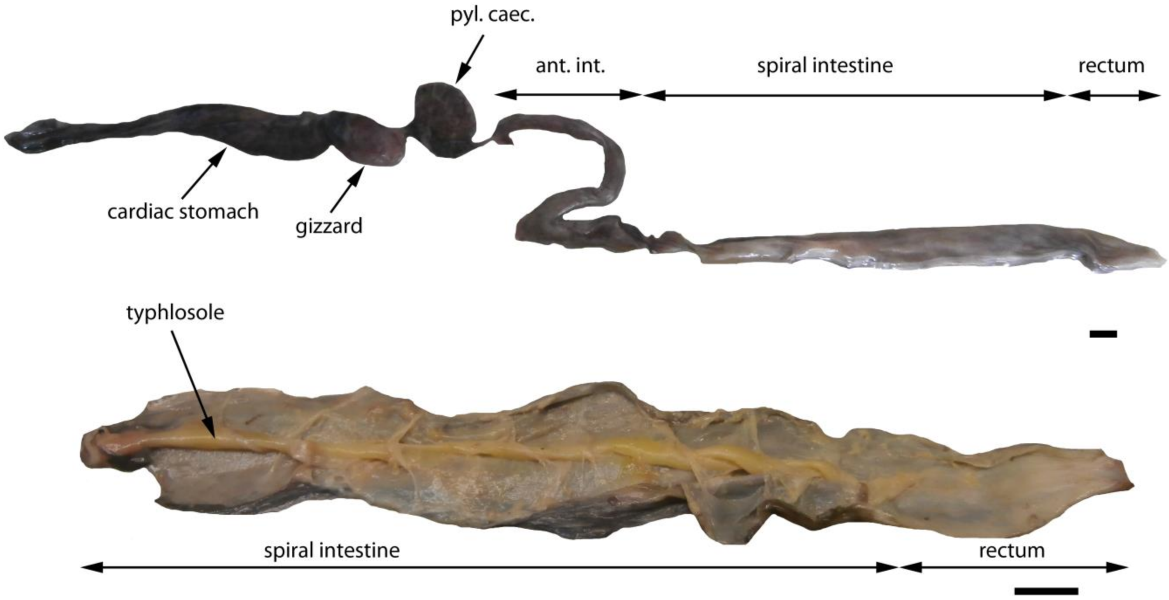

Supplementary Figure S5│ *Polyodon spathula* (VIMS 12227) dissected GIT in lateral (top left) and ventral (bottom left) views and dissected spiral intestine of the same specimen (right), for comparison. Scale bars equal 1 cm.

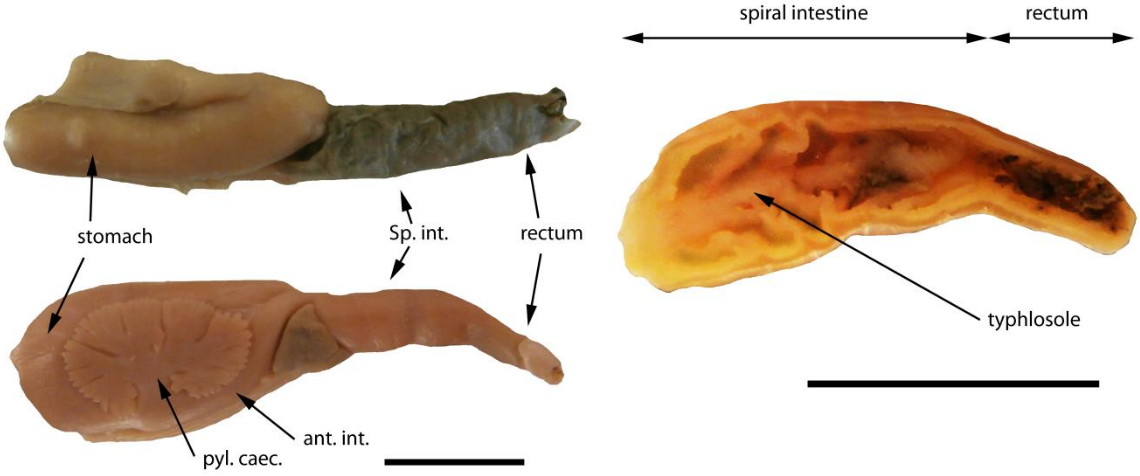

Supplementary Note 1, on the maturity of PIMUZ T 59

We consider the examined specimen of †*S. paucitrichus* (PIMUZ T 59) to be a mature individual. The developed gonopodium, the ossifications ventral to the notochord in the abdominal region and the ossified proximal and distal radials of the unpaired fins are all in support of this view (EEM and TA pers. obs.). The validity of †*S. paucitrichus* as a species as well as the attribution of various specimens to this taxon should be revisited[1].

Supplementary Note 2, on the tree used in the PGLS analysis.

We used Mesquite[2] to construct a composite tree based on an existing molecular tree of living elasmobranchs[3]. We manually added the extant actinopterygians sensu Near et al.[4] and the sarcopterygians following the consensus presented by Nelson[5]. The placement of fossil taxa is as follows: †*Saurichthys* sensu Gardiner et al.[6]; †*Liodesmus* and †*Amblysemius*, both grouped under the "superfamily" †Caturoidea, were placed as sister taxa on the *Amia* clade, following Grande and Bemis[7]; †*Macrosemius* (†Macrosemiidae) and †*Pericentrophorus* (†Semionotidae) were placed as sister taxa on the *Lepisosteus* clade following López-Arbarello[8]; †*Eurycormus* and †*Asthenocormus* were placed on the teleost stem sensu Arratia[9].

“Order”	Species	Maximum TL (m)	Maximum turn count	References
Heterodontiformes	*Heterodontus mexicanus*	0.7	7	10,11
Heterodontiformes	*Heterodontus portujacksoni*	1.65	7	10,11
Heterodontiformes	*Heterodontus zebra*	1.25	9	10,11
Heterodontiformes	*Heterodontus galeatus*	1.52	7	10,11
Heterodontiformes	*Heterodontus francisci*	1.22	7	10,11
Lamniformes	*Odontaspis ferox*	4.5	32	10,11
Lamniformes	*Odontaspis noronhai*	3.6	32	10,11
Lamniformes	*Carcharias taurus*	3.2	32	10,11
Lamniformes	*Mitsukurina owstoni*	3.84	19	10,11
Lamniformes	*Pseudocarcharias kamoharai*	1.1	27	10,11
Lamniformes	*Megachasma pelagios*	5.49	24	10,11
Lamniformes	*Alopias pelagicus*	3.65	40	10,11
Lamniformes	*Alopias superciliosus*	4.61	45	10,11
Lamniformes	*Alopias vulpinus*	5.73	34	10,11
Lamniformes	*Cetorhinus maximus*	10	51	10,11
Lamniformes	*Carcharodon carcharias*	6	55	10,11
Lamniformes	*Isurus oxyrinchus*	4.08	54	10,11
Lamniformes	*Isurus paucus*	4.17	54	10,11
Lamniformes	*Lamna ditropis*	3.05	41	10,11
Lamniformes	*Lamna nasus*	3.7	41	10,11
Orectolobiformes	*Parascyllium colare*	0.87	9	10,11
Orectolobiformes	*Brachaelurus waddi*	1.22	11	10,11
Orectolobiformes	*Brachaelurus cocloughi*	0.75	11	10,11
Orectolobiformes	*Eucrossorhinus dasypogon*	1.25	33	10,11
Orectolobiformes	*Orectolobus maculatus*	3.2	25	10,11
Orectolobiformes	*Orectolobus ornatus*	2.88	23	10-12
Orectolobiformes	*Orectolobus parvimaculatus*	0.88	28	10,11,13
Orectolobiformes	*Orectolobus halei*	2	32	10-12
Orectolobiformes	*Orectolobus floridus*	0.75	25	10,11,13
Orectolobiformes	*Orectolobus hutchinsi*	1.49	28	10,11,14
Orectolobiformes	*Chiloscyllium griseum*	0.77	19	10,11
Orectolobiformes	*Chiloscyllium hasseltii*	0.61	15	10,11
Orectolobiformes	*Chiloscyllium indicum*	0.65	15	10,11
Orectolobiformes	*Chiloscyllium plagiosum*	0.95	17	10,11
Orectolobiformes	*Chiloscyllium punctatum*	1.05	20	10,11
Orectolobiformes	*Stegostoma fasciatum*	3.54	18	10,11
Orectolobiformes	*Ginglimostoma cirratum*	4.3	17	10,11
Orectolobiformes	*Nebrius ferrugineous*	3.2	24	10,11
Orectolobiformes	*Pseudoginglymostoma brevicaudatum*	0.75	15	10,11
Orectolobiformes	*Rhincodon typus*	21	74	10,11
Carcharhiniformes	*Apristurus profundorum*	0.54	10	10,15
Carcharhiniformes	*Apristurus ampliceps*	0.85	11	10
Carcharhiniformes	*Scyliorhinus canicula*	1	11	10,16

Carcharhiniformes	*Scyliorhinus capensis*	1.22	11	10,16
Carcharhiniformes	*Scyliorhinus retifer*	0.47	11	10,16
Carcharhiniformes	*Scyliorhinus stellaris*	1.62	11	10,16
Carcharhiniformes	*Cephaloscyllium ventriosum*	1	12	10,16
Carcharhiniformes	*Poroderma africanum*	1.01	13	10,16
Carcharhiniformes	*Poroderma pantherinum*	0.84	13	10,16
Carcharhiniformes	*Asymbolus analis*	0.61	9	10,16
Carcharhiniformes	*Galeus arae*	0.36	14	10,16
Carcharhiniformes	*Galeus sauteri*	0.38	14	10,16
Carcharhiniformes	*Galeus polli*	0.3	14	10,16
Carcharhiniformes	*Galeus melastomus*	0.9	14	10,16
Carcharhiniformes	*Galeus murinus*	0.63	14	10,16
Carcharhiniformes	*Halaelurus lineatus*	0.56	10	10,16
Carcharhiniformes	*Halaelurus natalensis*	0.47	10	10,16
Carcharhiniformes	*Halaelurus buergeri*	0.49	10	10,16
Carcharhiniformes	*Haploblepharus edwardsii*	0.6	8	10,16
Carcharhiniformes	*Holohalaelurus regani*	0.61	7	10,16
Carcharhiniformes	*Parmaturus xaniurus*	0.45	8	10,16
Carcharhiniformes	*Eridacnis*	2	8	10,16
Carcharhiniformes	*Gollum attenuatus*	1.07	11	10,16
Carcharhiniformes	*Pseudotriakis microdon*	2.95	17	10,16
Carcharhiniformes	*Leptocharias smithii*	0.77	16	10,16
Carcharhiniformes	*Furgaleus macki*	1.6	7	10,16
Carcharhiniformes	*Galeorhinus galeus*	1.93	5	10,16
Carcharhiniformes	*Hemitriakis japanica*	1.2	8	10,16
Carcharhiniformes	*Hemitriakis leucoperiptera*	0.96	7	10,16
Carcharhiniformes	*Hypogaleus hyugaensis*	1.27	6	10,16
Carcharhiniformes	*Iago omanensis*	0.37	5	10,16
Carcharhiniformes	*Iago garricki*	0.75	6	10,16
Carcharhiniformes	*Mustelus asterias*	1.4	8	10,16
Carcharhiniformes	*Mustelus californicus*	1.16	8	10,16
Carcharhiniformes	*Mustelus canis*	1.5	9	10,16
Carcharhiniformes	*Mustelus henlei*	1	8	10,16
Carcharhiniformes	*Mustelus lenticulatus*	1.25	8	10,16
Carcharhiniformes	*Mustelus lunulatus*	1.7	9	10,16
Carcharhiniformes	*Mustelus manazo*	2.2	9	10,16
Carcharhiniformes	*Mustelus norrisi*	1.1	9	10,16
Carcharhiniformes	*Scylliogaleus quecketti*	0.89	7	10,16
Carcharhiniformes	*Triakis megalopterus*	1.42	6	10,16
Carcharhiniformes	*Triakis scyllium*	1.5	8	10,16
Carcharhiniformes	*Triakis semifasciata*	1.98	8	10,16
Carcharhiniformes	*Hemipristis elongatus*	2.4	6	10,16
Carcharhiniformes	*Hemigaleus microstoma*	1.14	6	10,16
Carcharhiniformes	*Paragaleus pectoralis*	1.4	6	10,16
Squaliformes	*Echinorhinus brucus*	3.1	16	10,17
Squaliformes	*Squalus acanthias*	1.6	13	10,17

Squaliformes	*Squalus suckleyi*	1.3	13	10,18
Squaliformes	*Squalus brevirostris*	0.6	9	10,19
Squaliformes	*Centrophorus granulosus*	1.7	14	10,17
Squaliformes	*Centrophorus lusitanicus*	1.6	13	10,17
Squaliformes	*Centrophorus squamosus*	1.64	14	10,17
Squaliformes	*Deania profundorum*	0.88	25	10,20
Squaliformes	*Centroscyllium fabricii*	0.7	10	10,17
Squaliformes	*Etmopterus gracilispinis*	0.35	11	10,21
Squaliformes	*Etmopterus bigelowi*	0.67	19	10,22
Squaliformes	*Etmopterus pussilus*	0.5	13	10,22
Squaliformes	*Etmopterus unicolor*	0.64	12	10,23
Squaliformes	*Etmopterus granulosus*	0.6	13	10,23
Squaliformes	*Centroscymnus coelolepis*	1.22	21	10,17
Squaliformes	*Centroscymnus owstoni*	1.2	15	10,17
Squaliformes	*Somniosus microcephalus*	7.3	34	10,17
Squaliformes	*Somniosus rostratus*	1.43	29	10,24
Squaliformes	*Somniosus pacificus*	4.4	37	10,24
Squaliformes	*Zameus squamulosus*	0.84	16	10,17
Squaliformes	*Oxynotus*	1.5	11	10,17
Squaliformes	*Isistius brasiliensis*	0.42	10	10
Squaliformes	*Squaliolus laticaudus*	0.28	13	10,17
Pristiophoriformes	*Pristiophorus nancyae*	0.62	7	10,25
Pristiophoriformes	*Pristiophorus lanae*	0.83	6	10,26
Squatiniformes	*Squatina*	2.44	12	10,17
Hexanchiformes	*Chlamydoselachus anguineus*	1.96	49	10,17
Hexanchiformes	*Heptranchias perlo*	1.39	22	10,17
Hexanchiformes	*Heptranchias griseus*	5.5	39	10,17
Hexanchiformes	*Heptranchias nakamurai*	1.8	28	10,17
Ceratodontiformes	*Neoceratodus forsteri*	1.7	9	10,27
Ceratodontiformes	*Protopterus annectens*	1	6	10,28
Polypteriformes	*Polypterus*	0.97	6	10,28
Polypteriformes	*Erpetoichthys calabaricus*	0.37	7	10,29
Acipenseriformes	*Polyodon spathula*	2.21	6	10,30, pers. obs.
Acipenseriformes	*Acipenser baerii*	2	8	10,31, pers. obs.
Acipenseriformes	*Acipenser transmontanus*	6.1	8	10,31, pers. obs.
Acipenseriformes	*Scaphirhynchus*	2	5	10,32
incerta	†*Saurichthys paucitrichus*	0.26	30	pers. obs.
Lepisosteiformes	*Lepisosteus osseus*	2	2	10,33, pers. obs.
†Semionotiformes	†*Pericentrophorus minimus*	0.04	8	34,35
†Semionotiformes	†*Macrosemius rostratus*	0.26	4	36,37
Amiiformes	*Amia calva*	1.09	4	10,38
Amiiformes	†*Amblysemius pachyurus*	0.26	17	37,39
Amiiformes	†*Liodesmus gracilis*	0.13	7	37,39
†Pachycormiformes	†*Asthenocormus titanius*	2.34	73	37,40
incerta	†*Eurycormus speciosus*	0.19	13	37,41

Supplementary Table 1 | Spiral valve turn count and maximum TLs of extant elasmobranch, sarcopterygian and actinopterygian taxa, and †*Saurichthys paucitrichus*, included in the regression analyses.

References

1 Maddison, W. P., & Maddison D. R (2008). Mesquite: A modular system for evolutionary analysis Version. 2.5. URL http://mesquiteproject.org/.

2 Naylor, G. J. *et al.* in *The biology of sharks and their relatives* (eds Carrier, J. C., Musick, J. A. & Heithaus, M. R.) 31-56 (CRC Press, 2012).

3 Near, T. J. *et al.* Resolution of ray-finned fish phylogeny and timing of diversification. *Proc. Natl. Acad. Sci. U.S.A.* **109**, 13698-13703 (2012).

4 Nelson, J. S. *Fishes of the world*. Fourth edn, (John Wiley & Sons, 2006).

5 Gardiner, B., Schaeffer, B. & Masserie, J. A review of the lower actinopterygian phylogeny. *Zool. J. Linn. Soc.* **144**, 511 - 525 (2005).

6 Grande, L. & Bemis, W. E. A comprehensive phylogenetic study of amiid fishes (Amiidae) based on comparative skeletal anatomy. An empirical search for interconnected patterns of natural history. *J. Vertebr. Paleontol. Mem.* **18**, 1-696 (1998).

7 López-Arbarello, A. Phylogenetic interrelationships of ginglymodian fishes (Actinopterygii: Neopterygii). *PLoS ONE* **7**, e39370 (2012).

8 Arratia, G. New teleostean fishes from the Jurassic of Southern Germany and the systematic problems concerning the 'pholidophoriforms'. *Paläontol. Z.* **74**, 113-143 (2000).

9 Maxwell, E. E., Romano, C., Wu, F. & Furrer, H. Two new species of *Saurichthys* (Actinopterygii: Saurichthyidae) from the Middle Triassic of Monte San Giorgio, Switzerland, with implications for character evolution in the genus. *Zool. J. Linn. Soc.* **173**, 887-912 (2015).

10 Froese, R. & Pauly, D. (2015). *Fishbase*. URL www.fishbase.org (accessed 5th of November 2015).

11 Compagno, L. J. V. *Sharks of the world. An annotated and illustrated catalogue of shark species known to date. Volume 2. Bullhead, mackerel and carpet sharks (Heterodontiformes, Lamniformes and Orectolobiformes). No. 1.* Vol. 2 (FAO, 2001).

12 Huveneers, C. Redescription of two species of wobbegongs (Chondrichthyes : Orectolobidae) with elevation of "*Orectolobus halei*" Whitley 1940 to species level. *Zootaxa*, 29-57 (2006).

13 Last, P. R. & Chidlow, J. A. Two new wobbegong sharks, *Orectolobus floridus* sp. nov. and *O. parvimaculatus* sp. nov. (Orectolobiformes: Orectolobidae), from southwestern Australia. *Zootaxa*, 49-67 (2008).

14 Last, P. R., Chidlow, J. A. & Compagno, L. J. V. A new wobbegong shark, *Orectolobus hutchinsi* n. sp. (Orectolobiformes: Orectolobidae) from southwestern Australia. *Zootaxa*, 35-48 (2006).

15 Rodríguez-Cabello, C., Pérez, M. & Bañon, R. Occurrence of *Apristurus* species in the Galicia bank seamount (NE Atlantic). *J. Appl. Ichthyol.* **30**, 906-915 (2014).

16 Compagno, L. J. V. *Sharks of the order Charcharhiniformes*. (Princeton University Press, 1988).

17 Ebert, D. A. & Stehmann, M. F. W. *Sharks, batoids and chimaeras of the north Atlantic*. Vol. 7 523 (Food and Agriculture Organization of the United Nations, 2013).

18 Ebert, D. A. *et al.* Resurrection and redescription of *Squalus suckleyi* (Girard, 1854) from the north Pacific, with comments on the *Squalus acanthias* subgroup (Squaliformes: Squalidae). *Zootaxa*, 22-40 (2010).

19 Qingwen, M. & Yuanding, Z. A study of the spiral valves of chinese cartilaginous fishes. *Acta Zool. Sin.* **31**, 277-284 (1985).

20 Coelho, R. & Erzini, K. On the occurrence of the arrowhead dogfish, *Deania profundorum* (Chondrichthyes: Squalidae) off southern Portugal, with a missing gill slit. *Cybium* **30**, 93-96 (2006).

21 Compagno, L. J. V., Ebert, D. A. & Smale, M. J. *Guide to the sharks and rays of southern Africa*. (Struik, 1989).

22 Shirai, S. & Tachikawa, H. Taxonomic resolution of the *Etmopterus pusillus* species group (Elasmobranchii, Etmopteridae), with description of E. bigelowi, n. sp. *Copeia* **1993**, 483-495 (1993).

23 Yano, K. First record of the brown lanternshark, *Etmopterus unicolor*, from the waters around New Zealand, and comparison with the southern lanternshark, *E. granulosus*. *Ichthyol. Res.* **44**, 61-72 (1997).

24 Yano, K., Stevens, J. D. & Compagno, L. J. V. A review of the systematics of the sleeper shark genus *Somniosus* with redescriptions of *Somniosus* (*Somniosus*) *antarcticus* and *Somniosus* (*Rhinoscymnus*) *longus* (Squaliformes: Somniosidae). *Ichthyol. Res.* **51**, 360-373 (2004).

25 Ebert, D. A. & Cailliet, G. M. *Pristiophorus nancyae,* a new species of sawshark (Chondrichthyes: Pristiophoridae) from southern Africa. *Bull. Mar. Sci.* **87**, 501-512 (2011).

26 Ebert, D. A. & Wilms, H. A. *Pristiophorus lanae* sp. nov., a new sawshark species from the western north Pacific, with comments on the genus *Pristiophorus* Müller & Henle, 1837 (Chondrichthyes: Pristiophoridae). *2013* **3752**, 15 (2013).

27 Rafn, S. & Wingstrand, K. G. Structure of intestine, pancreas, and spleen of the australian lungfish, *Neoceratodus forsteri* (Krefft). *Zool. Scripta* **10**, 223-239 (1981).

28 Jollie, M. *Chordate morphology*. (Rheinhold publishing corporation, 1968).

29 Purser, G. L. IV.—*Calamoichthys calabaricus* J. A. Smith. Part i. The alimentary and respiratory systems—concluded. *Earth Environ. Sci. Trans. R. Soc. Edinb.* **56**, 89-101 (1929).

30 Weisel, G. F. Anatomy and histology of the digestive system of the paddlefish (polyodon spathula). *J. Morph.* **140**, 243-255 (1973).

31 Buddington, R. K. & Christofferson, J. P. Digestive and feeding characteristics of the chondrosteans. *Environ. Biol. Fish.* **14**, 31-41 (1985).

32 Weisel, G. F. Histology of the feeding and digestive organs of the shovelnose sturgeon, *Scaphirhynchus platorynchus*. *Copeia* **1979**, 518-525 (1979).

33 Macallum, A. B. Alimentary canal and pancreas of *Acipenser*, *Amia*, and *Lepidosteus*. *J. Anat. Physiol.* **20**, 604-636 (1886).

34 Gall, J. C., Grauvogel, L. & Lehman, J. P. Les poissons fossiles de la collection Grauvogel-Gall. *Ann. paléontol. (Vertébrés)* **60**, 129-147+i-x tables (1974).

35 Jörg, E. Fischfunde im oberen Buntsandstein (Untertrias) von Karlsruhe-Durlach. *Zeits. Deuts. Geol. Ges.* **121**, 105-110 (1970).

36 Bartram, A. W. H. The macrosemiidae, a mesozoic family of holostean fishes. *Bull. Br. Mus. (Nat. Hist.), Geol.* **29**, 137-234 (1977).

37 Neumayer, L. Vergleichend anatomische untersuchungen über den darmkanal fossiler fische. *Abh. Bayer. Akad. Wiss.* **29**, 1-28 (1919).

38 Hilton, W. A. On the intestine of *Amia calva*. *Am. Nat.* **34**, 717-735 (1900).

39 Lambers, P. H. The halecomorph fishes *Caturus* and *Amblysemius* in the lithographic limestone of Solnhofen (Tithonian), Bavaria. *Geobios* **27, Supplement 1**, 91-99 (1994).

40 Liston, J. in *Mesozoic fishes 4. Homology and phylogeny* (eds Arratia, G., Schultze, H.-P. & Wilson, M. V. H.) 181-197 (Verlag Dr. Friedrich Pfeil, 2008).

41 Arratia, G. & Schultze, H.-P. *Eurycormus - Eurypoma,* two Jurassic actinopterygian genera with mixed identity. *Foss. Rec.* **10**, 17-37 (2007).
